# Supplementary material for: Enterovirus D68 seroepidemiology in Taiwan, a cross sectional study from 2017
Source: PLoS One. 2020 Mar 10;15(3):e0230180. doi: 10.1371/journal.pone.0230180 (PMC7064212; doi:10.1371/journal.pone.0230180)
Supplement: Attachment — Submitted filename: Response to reviewers.docx [file pone.0230180.s004.docx]

Point-by-point response

Dong-Yan Jin

Academic Editor

PLOS ONE

To enhance the reproducibility of your results, we recommend that if applicable you deposit your laboratory protocols in protocols.io, where a protocol can be assigned its own identifier (DOI) such that it can be cited independently in the future. For instructions see: <http://journals.plos.org/plosone/s/submission-guidelines#loc-laboratory-protocols>

Response:

We have deposited our laboratory protocols on the website protocols.io. For details, please see <https://www.protocols.io/view/the-ev-d68-neutralizing-antibody-test-baknicve>.

Reviewer #1:

The logic of data analysis is wrong, hence, the results maybe not right. To analyse the risk factor for the infection, firstly, the univariate analysis maybe applied, if the P<0.2, the factor is entered into multivariate analysis, if P>=0.2, the factor cannot be entered into multivariate analysis. Only in multivariate analysis, the dummy variable be needed and meanly meaningful. Hence, please reanalyze data (for instance, table 2).
The reference may be useful for you. ”Risk factors for Blastocystis infection in HIV/AIDS patients with highly active antiretroviral therapy in Southwest China”.

Response:

We used the analytical method you suggested. The factors with *p*-values <.2 in the univariate analysis were selected for multivariate analysis. In our study, we identified age, region, size of household, number of siblings and kindergarten/daycare attendance as significant factors in the univariate analysis. There was a significant correlation between household size and the number of siblings (Spearman correlation, r_s_=0.36, *p*-value<0.0001), so we selected household size for multivariate analysis. In the multivariate analysis, we identified age, household size, and kindergarten/daycare attendance as risk factors for EV-D68 seropositivity. Table 2 was modified, as shown in the revised manuscript. We also added some discussion regarding household size: Children with a larger household size had a significantly higher seropositive rate, implying that a larger household size may be associated with a higher risk for EV-D68 household transmission. We hope the reanalysis better demonstrates the characteristics of our study.

Reviewer #2: The study by Lee et al describes a serosurvey of EV-D68 neutralising antibodies among the Taiwanese population as well as trying to identify factors associated with seropositivity. The study was well-designed and the cohort size was substantial. Results demonstrate a remarkably high overall seropositivity in an age-dependent manner similar to results of other studies.

Importantly, the manuscript should be proof read by a native English speaker as there are many grammatical or inaccuracies throughout the manuscript. This would improve the readability and clarity of the manuscript considerably.

Response:

Thank you very much for your comments. The manuscript has been carefully proofread by a native English speaker. The editing certificate was in the supplementary materials.

- Table 1: in the age category, please add ages to the adult women/men category.

Response: We have added ages to the adult women/men category in Table 1

- Table 3: The study in the Netherlands investigated neutralizing Abs against two EV-D68 strains, please indicate to which data (Fermon strain or B3 clinical) is referred to in this table. It would be helpful to indicate for each study, which D68 strain was used.

Response: We added the strain used in the Dutch study and other studies.

- The data from table 3 could also be presented in a chart allowing for an easier comparison of the seropositivity

Response: Thank you very much for your suggestion. We would like to make a chart but could not complete it due to different age groups, different viral strains and too many countries in all the studies. Therefore, we are sorry to keep it as Table.

- Fig. 1: I would suggest bar graphs instead of a connected line. Since the average for all investigated areas is given, please show also the mean +- SD.

Response: According to your suggestion, we switched to bar graphs including mean +- SE, as shown in the revised manuscript.
